# Supplementary material for: Smartcrystals for Efficient Dissolution of Poorly Water-Soluble Meloxicam
Source: Pharmaceutics. 2022 Jan 21;14(2):245. doi: 10.3390/pharmaceutics14020245 (PMC8879336; doi:10.3390/pharmaceutics14020245)
Supplement: Supplementary file 1 [file pharmaceutics-14-00245-s001.zip › pharmaceutics-1510423-supplementary.pdf]

pharmaceutics

Supplementary Materials: Smartcrystals for Efficient Dissolution of Poorly Water-Soluble Meloxicam

Rita Ambrus, Areen Alshweiat, Piroska Szabó-Révész, Csilla Bartos and Ildikó Csóka

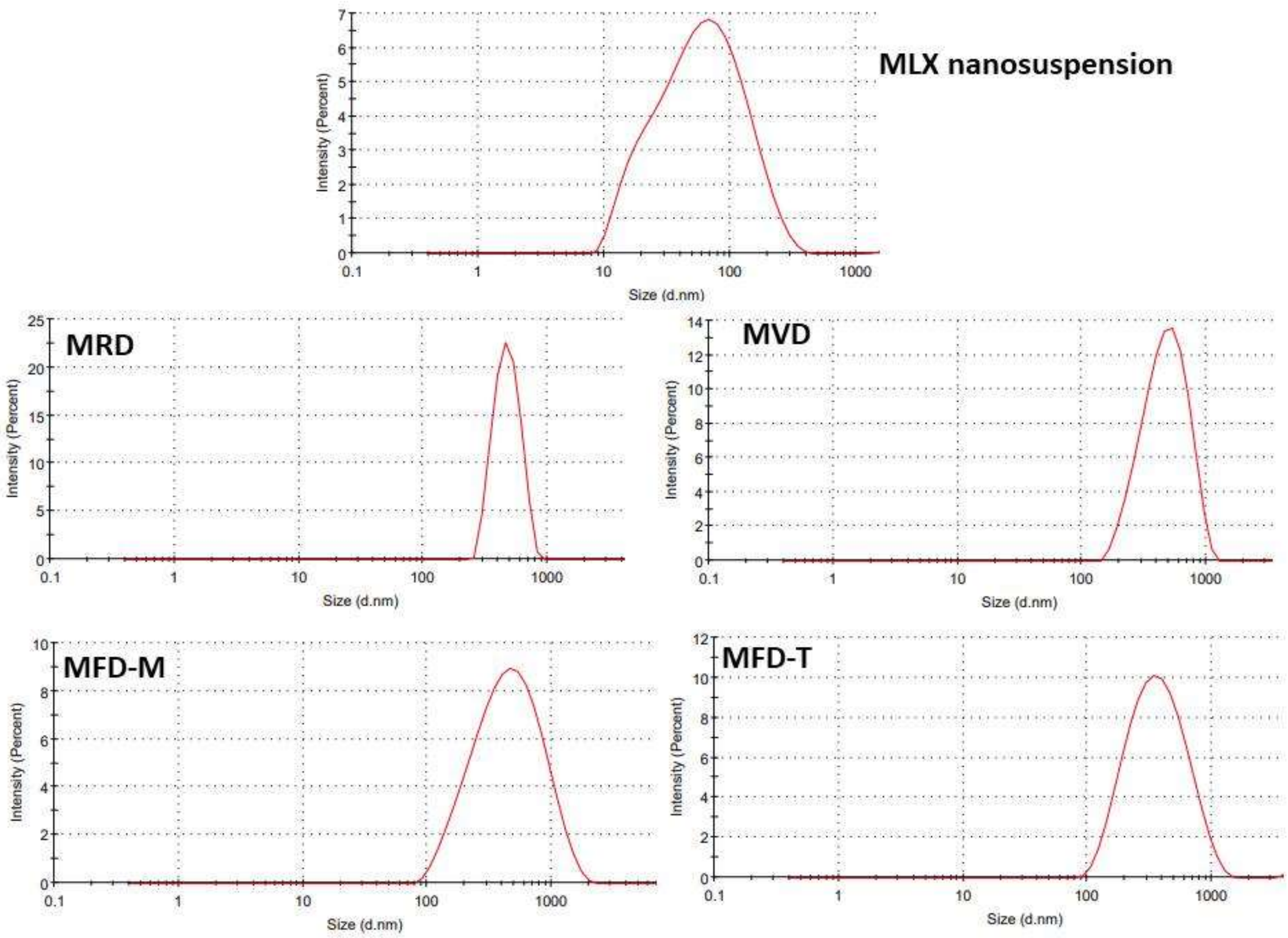

Figure S1. MPS of MLX nanocrystals after redistribution in water.

Pharmaceutics **2022**, *14*, 245. https://doi.org/10.3390/pharmaceutics14020245

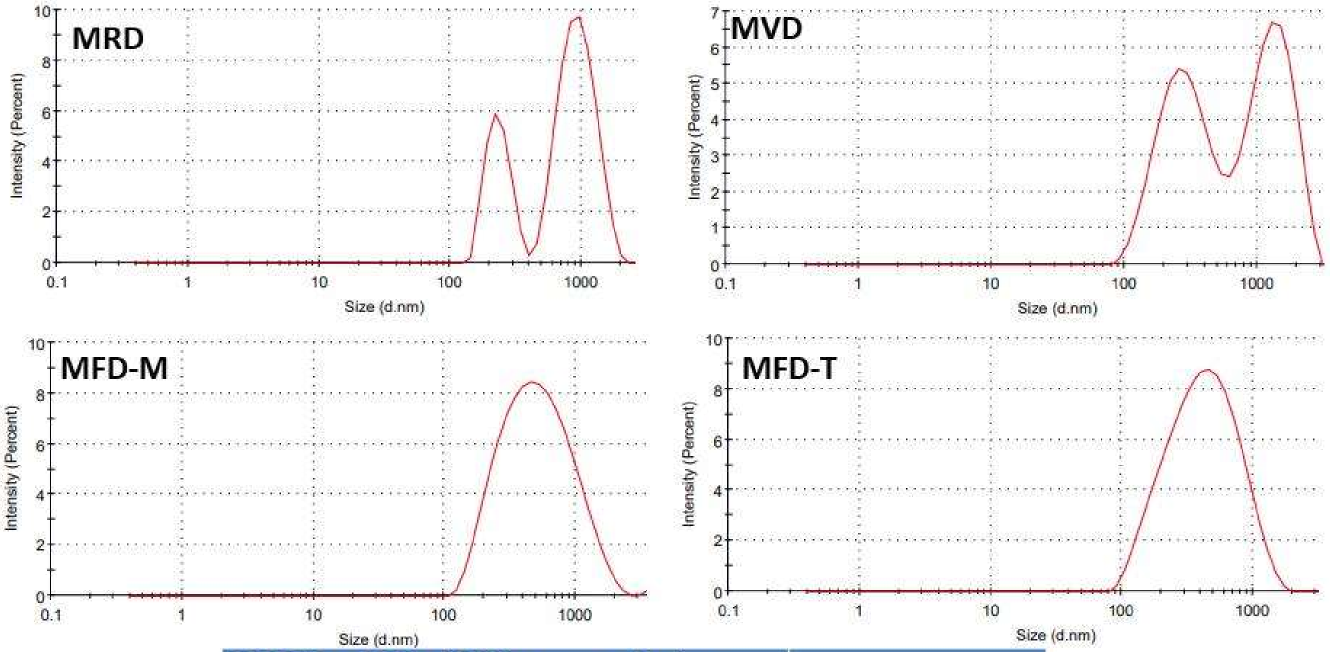

MPS, ZP and PDI of MLX nanocrystals after redistribution in water (1 month storage)		
Sample	MPS (nm)	ZP
MRD	804.9 ± 32.12	-12.6
MVD	653 ± 17.21	-16.8
MFD-M	445.57 ± 12.56	-18.6
MFD-T	357.42 ± 2.47	-22.9

Figure S2. MPS, ZP and PDI of MLX nanocrystals after redistribution in water (1-month storage).

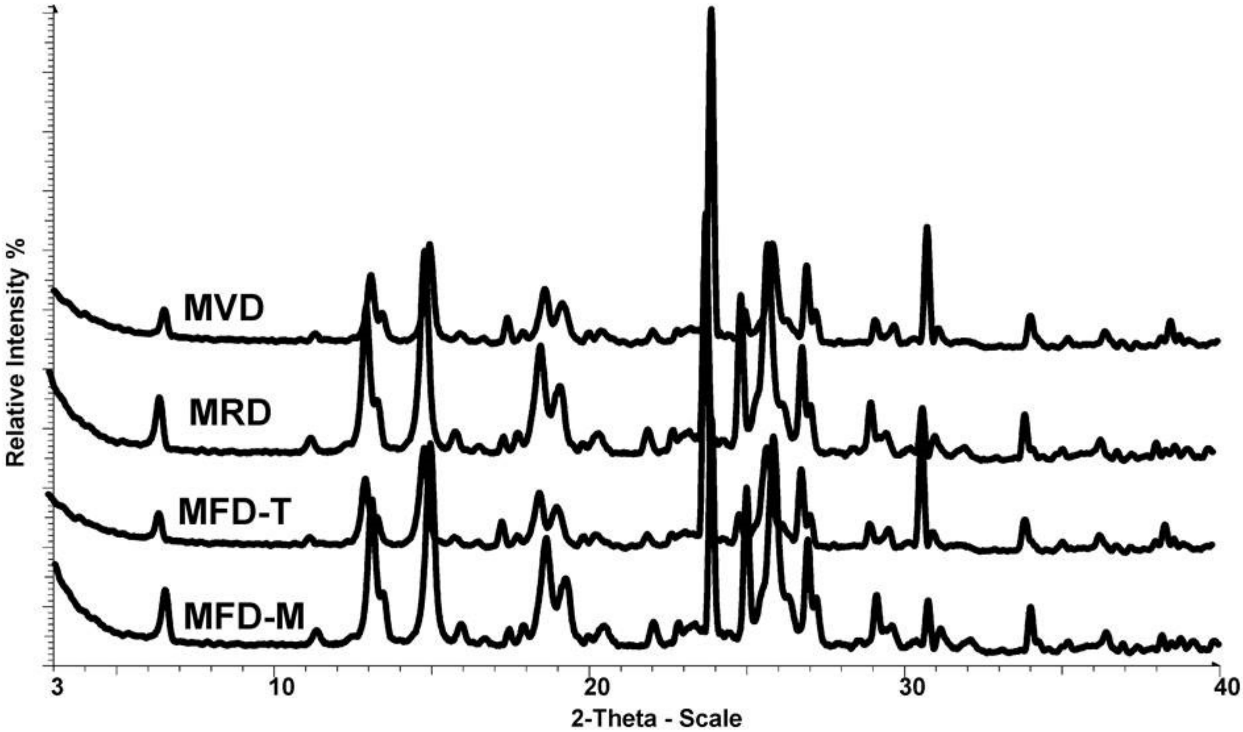

Figure S3. XRPD patterns of the samples after dissolution to recrystallized the samples from the media.
